# The prevalence of cervical abnormalities: Comparison of youth with perinatally acquired HIV and older women in Botswana

**DOI:** 10.4102/sajhivmed.v24i1.1455

**Published:** 2023-03-28

**Authors:** Thabo Phologolo, Mogomotsi Matshaba, Bathusi Mathuba, Keboletse Mokete, Ontibile Tshume, Elizabeth Lowenthal

**Affiliations:** 1 Department of Family Medicine and Public Health, Faculty of Medicine, University of Botswana, Gaborone, Botswana; 2 Botswana-Baylor Children’s Clinical Centre of Excellence, Gaborone, Botswana; 3 Department of Pediatrics, Section of Retrovirology, Baylor College of Medicine, Houston, United States of America; 4 Departments of Pediatrics and Epidemiology, Biostatistics and Informatics, Perelman School of Medicine, University of Pennsylvania, Philadelphia, United States of America; 5 Global Health Center, Children’s Hospital of Philadelphia, Philadelphia, United States of America

**Keywords:** perinatal HIV, young women, visual inspection with acetic acid, cervical cancer screening, Africa

## Abstract

**Background:**

Cervical cancer burden and prevalence of precursor lesions is unknown among young women living with HIV in high prevalence settings. Current cervical cancer screening guidelines in resource-limited settings with high HIV prevalence typically exclude adolescents and young women. After observing two cases of advanced cervical cancer among young women with perinatally acquired HIV, a pilot screening programme was established in Botswana.

**Objectives:**

To compare the prevalence of cervical abnormalities in young women with perinatally acquired HIV with women aged 30–49 years, regardless of HIV status.

**Method:**

We conducted a cross-sectional study of 30–49-year-old women who had visual inspection with acetic acid screening through the Botswana public sector programme, and youth (aged 15–24 years) with perinatally acquired HIV, at a single referral site between 2016 and 2018. We describe the prevalence of cervical abnormalities in each group as well as the crude prevalence ratio.

**Results:**

The prevalence of cervical abnormalities in women 30–49 years of age was 10.9% (95% confidence interval [CI]: 10.4, 11.4), and 10.1% (95% CI: 4.7, 18.3) for youth. The crude prevalence ratio was 1.07 (95% CI: 0.58, 2.01).

**Conclusion:**

Inclusion of youth living with HIV in cervical cancer screening services should be considered in settings with a high prevalence of HIV and cervical cancer.

**What this study adds:** This study shows that the prevalence of cervical abnormalities amongst youth with perinatally acquired HIV may be comparable to that of older women in high-burden HIV settings.

## Background

Cervical cancer burden and prevalence of precursor lesions is unknown among young women with perinatally acquired HIV, living in high HIV prevalence settings. Young women are generally excluded from cervical cancer screening programmes in current guidelines in high HIV prevalence settings, which prioritise women aged 30–49 years. This approach is contrary to recommendations from other settings for increased frequency of cervical cancer screening and follow-up amongst people living with HIV due to faster progression of disease.^1^ Targeted vaccination with the human papillomavirus (HPV) vaccine is recommended as data suggest that this decreases the risk of developing cervical cancer, although it does not remove the need for screening.^2^ Limited data from other settings^3,4,5^ and anecdotal evidence suggested that the burden of cervical abnormalities might be substantial in young women with perinatally acquired or early childhood HIV infection.

Several biological processes have been suggested to explain this phenomenon. HIV increases HPV transmission. CD4+ T-lymphocytes play a central role in the control of established HPV infections. Therefore, HIV-related immune suppression facilitates viral persistence,^1^ and persistent HPV infection is associated with developing cancer.^6^ Faster progression of cervical abnormalities has been found in the context of HIV.^7^ In youth who were perinatally infected with HIV, the immaturity of the immune system at the time of HIV acquisition, the longevity of immune dysregulation and the frequency of challenges with medication adherence suggests that sexually active youth with perinatally acquired HIV may have similar or higher vulnerability to advanced cervical abnormalities than sexually active older women. However, as an association between antiretroviral therapy initiation at low CD4 counts and faster progression of cervical abnormalities has been shown, women who become infected later in life and start treatment at low CD4 counts may in some instances be at greater progression risk than perinatally infected adolescents who are commenced on antiretroviral therapy early.^8^

To test our hypothesis that young women with perinatally acquired HIV have similar prevalence of cervical abnormalities to women of 30–49 years, we compare the prevalence of cervical abnormalities between the women evaluated through the national screening programme in Botswana and a pilot project for screening adolescents and young women living with HIV at the Botswana-Baylor Children’s Clinical Centre of Excellence (BBCCCOE), a centre focused on the care and treatment of children and youth living with HIV. Both programmes implemented visual inspection with acetic acid (VIA) screening, using the same equipment, supplies, trainings and Ministry of Health (MoH)-supported mentorship.

## Methods

### Setting

Based on stakeholder consensus and international evidence-based recommendations as provided by the World Health Organization, Botswana began implementation of a multipronged cervical cancer prevention and control programme in 2012. The prevention aspect focused on HPV vaccines for girls 9–13 years, while secondary prevention in the form of screening and early treatment targeted women 30–49 years of age. Women outside this age range typically are only screened if symptomatic.^5,6^ Data from the government screening programme includes women screened in both primary and tertiary care health facilities offering VIA countrywide. Youth data were obtained from a single tertiary care site, the BBCCCOE, a public-private partnership comprehensive free HIV clinic, located in Gaborone, Botswana. As it is the largest specialised paediatric and adolescent HIV clinic in Botswana, individuals come from all parts of the country and represent all socioeconomic strata. The clinic has 2394 registered clients living with HIV, of whom 671 are female between the ages of 15 and 24 years. The site treats children and youth, regardless of mode of HIV acquisition. However, most are enrolled as infants and young children, with documented HIV-positive mothers, which strongly suggests that they may have been infected perinatally. After observing two cases of cervical cancer among perinatally HIV-positive youth, the BBCCCOE instituted a pilot cervical abnormalities screening programme in 2017 for sexually active asymptomatic youth in their clinical cohort. The BBCCCOE cervical cancer screening clinic is primarily a VIA site, with Pap smears done either as a second-level test for inconclusive VIA test or as sole screening where it is anatomically or medically impossible or inappropriate to do VIA screening.

### Participants and definitions

Data were included for: (1) women 30–49 years of age, regardless of HIV status, who were screened with VIA for the first time between 2016 and 2018 through the national screening programme; and (2) adolescents and young adults < 24 years of age (‘youth’) with perinatally acquired HIV screened with VIA at the BBCCCOE cervical cancer screening clinic for the first time in 2017 and 2018. We defined perinatal HIV infection as HIV infection that was thought to have resulted from the transmission of HIV from the mother to her child during pregnancy, labour and delivery, or breastfeeding.^6^

Our primary outcome of cervical abnormality was defined as a positive VIA or positive VIA-suspected cervical cancer The national guidelines define a positive VIA as a change in the cervix upon application of the acetic acid, resulting in well-defined, opaque-acetowhite lesions in the transformation zone close to the squamocolumnar junction or to the external orifice, or the entire cervix turning acetowhite.^9^ A positive VIA-suspected cervical cancer is reported as a change whereby one or many cervical growths turn acetowhite.^10^ For youth testing at BBCCCOE, we report on an abnormal Pap smear test, irrespective of whether VIA test was done. An abnormal Pap smear test was considered a cervical abnormality if the finding was low-grade squamous intraepithelial lesion or high-grade squamous intraepithelial lesion. Atypical squamous cells of undetermined significance were not classified as a cervical abnormality.

### Data source

We used the National Cervical Cancer Screening Database to extract data for women screening for cervical cancer receiving VIA for the first time. This database is housed in the Botswana MoH and is disaggregated by age groups, HIV status (positive, negative, unknown), VIA test results (VIA positive; VIA positive – suspected cervical cancer; VIA negative), and procedures or referrals done in the event of a positive VIA test (cryotherapy, Loop Electrosurgical Excision Procedure, colposcopy). The MoH data are reported at the facility-level. Individual-level data are not available for the national cohort. The BBCCCOE registers and patient electronic data were used to extract and abstract variables for individual youth living with HIV who were included in the screening programme.

### Inclusion/exclusion criteria

Because the national screening guidelines do not differ based on HIV status, we included data from all women between the ages of 30–49 who were having their first VIA, regardless of HIV status.

For the BBCCCOE sample, all data were from HIV-positive youth aged 16–24 years who were having their first VIA. Screening at the BBCCCOE was offered to sexually active females patients ≥ 16 years of age.

### Sample size and power

We used exhaustive sampling for both groups. We estimated that there would be 120 youth screened at BBCCCOE and approximately 6000 30–49-year-old women in the public programme during the sampling period.

Considering the reported prevalence of positive VIA among women of mixed HIV status in the region (30.5% among farm workers and sex workers in South Africa’s Limpopo province,^11^ 28% in general public in Zambia, 12.4% in general public in Malawi^12^), we calculated detectable differences across the plausible range of 15% – 25% with positive VIA in the 30–49-year age group in Botswana. With the available sample size, we calculated that we would have 80% power to detect a prevalence ratio for cervical abnormalities less than 0.6 or greater than 1.5 with a two-sided alpha level of 0.05 for the dichotomous comparison of cervical abnormalities/no cervical abnormalities.

### Data collection

All data were de-identified and either electronically or manually imported into Excel files by trained and authorised staff of the respective organisations. All MoH data were downloaded from the national database which is populated at the individual clinic level prior to being compiled centrally. Data were manually extracted from the BBCCCOE medical records, both electronic and paper-based, by clinic research nurses.

We evaluated for missing and out of range data for the two data sets. Data from the MoH were double-checked by the study Principal Investigator and an MoH data officer against the submitted reports from the respective facilities. All data from the BBCCCOE were double-checked for accuracy against the primary source by the study Principal Investigator, the study nurse, and the nurse in charge of the cervical cancer screening programme.

To protect study participants from the youth sample, medical record numbers were replaced with unique study numbers. Dates of birth were converted into the age at the time of the VIA evaluation.

Data on HIV status (positive/negative/unknown) were reported in the national database. For the perinatally infected cohort, data were collected regarding whether they had ever used hormonal contraception, age of sexual debut, parity, CD4+ T-lymphocyte count (CD4 count) nadir and most recent CD4 count, most recent HIV viral load, and smoking history. HIV viral load was considered to be undetectable for values less than 400 copies/mL, as per Botswana guidelines. The most recent viral loads for all youth were within 6 months of index VIA.

### Statistical analysis

Data were imported into the Stata IC/14.2^®^ (Stata Corporation, College Station, Texas, United States) statistical software package for analysis. For our primary analysis, we used the composite endpoint of cervical abnormalities versus no cervical abnormalities. The proportion of cervical abnormalities versus no cervical abnormalities was compared between women 30–49 years of age in the national testing group and BBCCCOE youth, using chi-squared test with the criterion for significance set as a *P*-value < 0.05. Mean and standard deviation are reported for parametric data and median (interquartile range [IQR]) for non-parametric data. We calculated the crude cervical abnormalities prevalence ratio and 95% confidence interval (CI) by dividing the cervical abnormalities prevalence of women 30–49 years by that of BBCCCOE youth.

## Results

A total of 14 819 women aged 30–49 years attended VIA screening clinics across the country for the first time between January 2016 and December 2018, in 23 of the total 27 health districts. Of the 92 youth who attended the BBCCCOE cervical cancer screening clinic, 85 of 92 (92.4%) were screened with VIA alone, three (3.3%) with Pap alone and four (4.4%) with both Pap and VIA.

For the BBCCCOE group, the median age in years for screening was 22 years (range: 17 to 24), and 18 years (range: 8 to 23) for sexual debut. The lowest age of sexual debut was reported in a case of sexual abuse. The median total number was 3 (range: 1 to 16; IQR: 2–5) for sexual partners and 0 (range: 0 to 3; IQR: 0–1) for parity. The median CD4 count was 644 cells/µL (IQR: 370.5–882). The HIV viral load was undetectable in 77 (83.7%) of participants. Twenty-six (29.9%) participants reported ever using hormonal contraception and six (6.6%) reported ever smoking. Characteristics of screened adults are further described in Table 1, and youth in the perinatally infected cohort in Table 2.

**TABLE 1:** Results of visual inspection with acetic acid in adult women (30–49 years of age) first-time testers (2016–2018).

Values	HIV+	HIV−	Unknown	Total
Number done	7444	5958	1417	14 819
Cervical abnormalities	1135	375	107	1617
Median prevalence (%)	15.2	6.3	7.6	10.9

+, positive; −, negative.

**TABLE 2:** Characteristics of perinatally HIV-positive youth screened with visual inspection with acetic acid.

Characteristic	Median	IQR	Number	%
Age in years	22	20–23	-	-
Age of sexual debut in years (*n* = 56)†	18	17–19	-	-
Lifetime sexual partners (*n* = 56)†	3	2–5	-	-
Parity (*n* = 58)†	0	0–1	-	-
Recent CD4 count	644	370.5–882	-	-
**Recent viral load detectable** ‡				
Yes	-	-	15	16.3
No	-	-	77	83.7
**History of smoking tobacco**				
Yes	-	-	6	6.6
No	-	-	50	54.3
Missing	-	-	36	39.1
**Hormonal contraceptive use**				
Yes	-	-	26	28.3
No	-	-	61	66.3
Missing	-	-	5	5.4

IQR, interquartile range.

† , Number included for each variable = 92 except where indicated.

‡ , HIV viral load was considered detectable when ≥ 400 copies/mL.

A total of 1617 women and nine youth had cervical abnormalities, giving a prevalence of cervical abnormalities of 10.9% (95% CI: 10.4, 11.4) for women 30–49 years of age and 10.1% (95% CI: 4.7, 18.3) for youth living with HIV. The crude prevalence ratio was 1.07 (95% CI: 0.58, 2.01). Of the seven Pap smears done amongst the youth, three were atypical squamous cells of undetermined significance, two low-grade squamous intraepithelial lesions, one negative and one not reported.

Cases of suspected cancer constituted 8.7% (95% CI: 7.3, 10.1) of cervical abnormalities amongst the older women, while no cases of suspected cancer were reported for youth. Amongst youth with cervical abnormalities, three (33.3%) had detectable viral loads while viral loads were detectable in 11 (13%) of those without cervical abnormalities. The median CD4 count amongst youth with cervical abnormalities was 342 cells/µL (IQR: 188.5–455.5), and 656 cells/µL (IQR: 450–905) for those without cervical abnormalities.

## Discussion

Our finding in this cross-sectional study of a group of 30–49-year-old women who received VIA screening in the public sector and perinatally HIV-positive youth at a single referral site suggests that the risk of cervical abnormalities of these two groups is similar. The overall prevalence of cervical abnormalities was lower in our cohorts (10.1%) compared with previously published samples from the region (15% – 30%).^13,14^ However, cervical cancer remains the leading cause of cancer in Botswana.^15^ Despite the fact that effective screening is available for women in the 30–49-year-old age bracket, relatively few patients presenting with invasive cervical cancer have ever accessed screening services.^16^ Furthermore, mortality rates are highest among women with cervical cancer who are also HIV-positive.^17^

Spontaneous improvement of cervical abnormalities is possible,^18^ and improvements in immunologic status have been associated with lower prevalence and slower progression of cervical abnormalities in women living with HIV.^19^ However, our study suggests that national policies that exclude youth from cervical cancer screening programmes likely come with a substantial risk of late cancer presentation for youth who are living with HIV. This may be particularly true for youth who were perinatally HIV-positive due to the longstanding nature of their HIV-related immune aberrations. Due to the comparable prevalence of cervical abnormalities among youth with perinatally acquired HIV and 30–49-year-old women who qualify for the national cervical cancer screening programme, expansion of youth-friendly cervical cancer screening and prevention interventions seems prudent. Policymakers need to consider their country’s unique disease epidemiology and ethical ideal of universal access and equity in prioritisation of recipients of their cervical cancer screening programmes. Advancing the international efforts to improve overall care for adolescents and young adults infected with HIV is a challenge that requires outside the box thinking by providers of health services.

This study was limited by the lack of individual-level data for the approximately 15 000 women screened through the national programme. The size of the individual-level data sample for the youth was relatively small since cervical cancer screening for younger women is not currently part of the public treatment programmes. Additionally, screening was done with VIA while the World Health Organization recommendation is that of using a DNA-based HPV assay.^19^ While we were interested in looking at the relationship between variables such as age at first sexual encounter, number of sexual partners, parity and tobacco use and cervical abnormalities in the perinatally HIV-positive cohort, the relatively small numbers of individuals in this sample precluded meaningful comparisons.

Our data suggest that cervical cancer screening and prevention services should be expanded to include youth living with HIV as a target population in high cervical cancer and HIV prevalence settings, such as Botswana. Future studies should evaluate the progression/regression of cervical abnormalities among youth living with HIV, including both those who were perinatally and behaviorally infected.
